# Increasing the detection distance of remote NMR using wireless inductive coupling coil

**DOI:** 10.1038/s41598-017-12854-x

**Published:** 2017-10-04

**Authors:** Mario Henrique M. Killner, Giancarlo Tosin, André S. Carvalho, Diego Firme Bernardes, Luiz Alberto Colnago

**Affiliations:** 1 Embrapa Instrumentação, Rua XV de Novembro 1452, São Carlos, SP 13560-970 Brazil; 2 Universidade Estadual de Londrina, Pr 445–km 380, Londrina, PR 86057-970 Brazil; 3 LMA Magnet Consultancy, Rua Filomeno Rispoli 509, 13564-200 São Carlos, São Paulo Brazil; 4 Instituto de Química de Sao Carlos, Universidade de São Paulo, Av. Trabalhador São-carlense 400, São Carlos, SP Brazil

## Abstract

Unilateral nuclear magnetic resonance (UNMR) spectrometers have been applied in a variety of fields such as petrochemistry, materials science, and process control^**1**^. In UNMR measurements the sample is placed outside of the UNMR sensor and the signal intensity is reduced almost exponentially as the sample-to-sensor distances increases. To expand the detection limits of remote UNMR sensors, wireless inductive coupling was proposed and tested. This strategy was proved to reduce signal attenuation due to sample detachment from sensor, resulting in an increase in detection distance by one order of magnitude (i.e., from few millimeters to few centimeters). This novel approach broadens the potential applications of UNMR sensors and opens new opportunities in several areas, from chemical to biomedical applications.

## Introduction

Nuclear magnetic resonance (NMR) spectroscopy and imaging have been among the most powerful analytical tools applied from atoms/molecules to large living organism, including human beings. Most of these applications are performed inside bulky and heavy magnets, being sample or organism dimensions restricted by the magnet’s bore or gap^1^. In order to bypass this limitation, several magnet and probe designs (e.g., opposite field and single-sided or unilateral magnets) have been developed for remote NMR detection. In these systems, the analyses are performed outside of the sensor (remote detection) so that sample dimension is not a limiting factor^1^. The first commercial application of this principle was in the petroleum industry, in which remote NMR sensors denote a standard tool for well-logging procedures to estimate oil, gas, and moisture contents, reservoir porosity, and many other parameters related to porous media^3^.

Encouraged by the use of permanent magnets, much smaller unilateral NMR sensors weighting few kilograms and accepting inhomogeneous and strong gradient fields for depth profile have been developed and used in the past two decades. These sensors first appeared in the scientific literature in 1995 along with the NMR-MOUSE^4^, a mobile surface scanner based on two small permanent magnets mounted on a horseshoe geometry. Thereafter, different permanent magnet geometries have been developed in order to increase the depth sensitivity range of such unilateral sensors. These new NMR surface explorers and depth profiling have been used to analyze samples in various fields (e.g., chemistry, physics, archeology, human heritage, and biomedical, materials, and environmental sciences) in different measurements, including remote monitoring of chemical reactions, viscosity, porosity, diffusion through materials, painting authenticity, and diseases (e.g., cancer), among many other applications^4–8^.

An inherent, unwanted characteristic of unilateral NMR (UNMR) devices is that the signal decays roughly exponentially with distance from sensor surface, limiting the detection distance to a few millimeters for small UNMR sensors. Numerous efforts have been proposed to increase the detection distance based on magnetic geometry^9,10^ and/or coil arrangements^11^. In this study, we demonstrate a simple, efficient strategy to expand the detection limits of small UNMR sensors based on wireless inductive coupling sensor (coil and tuning capacitor). Inductive coupling coils have been used to enhance signal-to-noise ratio (SNR) in high-resolution solid-state NMR and magnetic resonance imaging^12–14^. This new configuration increases the detection limit by one order of magnitude, from a few millimeters to a few centimeters.

## Results

### Secondary coil coupling to the unilateral NMR device

**Figure 1:**
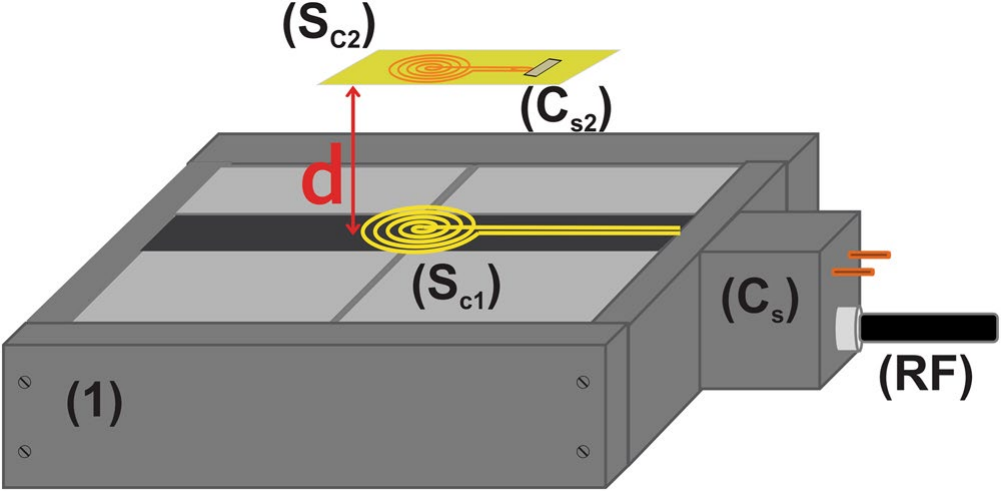
Apparatus used in this work, in which: (**1**) UNMR sensor; (**Sc**_**1**_) primary surface coil used on UNMR probe; (**Sc**_**2**_) secondary coil inductive coupled to **Sc**_**1**_ and located at a distance **d** from **Sc**_**1**_; (**C**_**s**_) aluminum box with the matching and tuning variable capacitors of **Sc**_**1**_ coil; (**C**_**s2**_) chip ceramic capacitor connected in shunt with **Sc**_**2**_; (**RF**) coaxial cable connecting the magnetic sensor to the spectrometer.

Fig. 1 illustrates the apparatus applied in this work. The secondary coil (**S**_**c2**_) is composed of a thin copper coil etched on a printed circuit board. This coil was tuned by soldering a chip capacitor onto it. The capacitor was chosen based on the primary coil frequency that maintained the so-called ‘over coupling’ regime wherein all the dissipated power in the primary coil is essentially dissipated in the secondary circuit. To reach as higher **SNR** values as possible, the coupled system was also matched to 50 Ω ^12–15^.

In a typical single-sided magnet, the coil is placed close to the magnet surface, as depicted by **S**_**C1**_ in Fig. 1. When the region to be analyzed is far from the **S**_**C1**_ coil, the signal induced by nuclear spin precession and relaxation usually has remarkably low intensity. A straightforward means of increasing signal intensity is by surrounding the sample region with a second coil **S**_**C2**_, which in turn is coupled by mutual-inductance to **S**_**C1**_.

**Figure 2:**
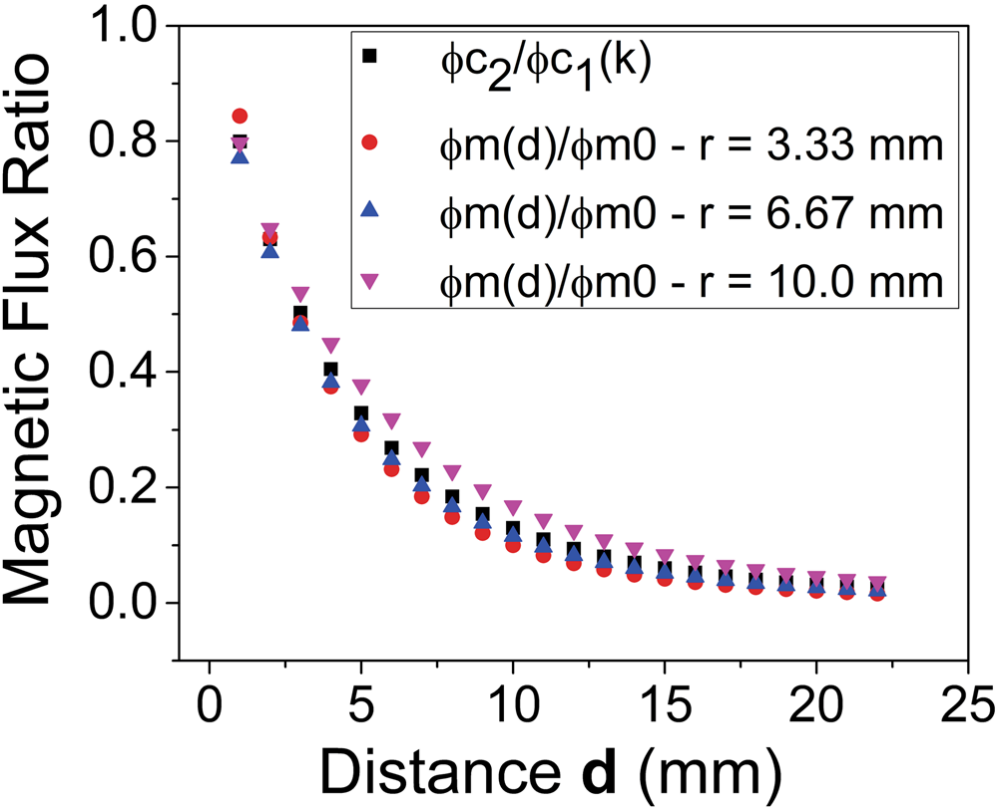
Magnetic Flux Ratio for three magnetized disks with different radii as well as the coupling (k) between the **S**_**C1**_ and **S**_**C2**_ coils.

Computational simulations were carried out using FEMM 4.2 software^**Y20**^ in order to estimate the relative magnetic flux in the **S**_**C1**_ and **S**_**C2**_ coils generated by the nuclear spins. Thus, homogenously narrow disks with width similar to the diameters of coil wire, magnetized perpendicularly to the coil surface, were positioned concentrically to the coil axis. The magnetic flux perpendicular to the coil surface Φ_*m*_(*d*) was then calculated as a function of the distance ***d*** from the coil to the magnetized region. Figure 2 presents Φ_*m*_(*d*) normalized by the flux ${{\rm{m}}}^{0}$, which corresponds to **d** = 0 mm, for three different magnetized disk diameters: 6.7, 13.3 and 20 mm.

Although single-sided magnets do not feature a homogeneous circular region, calculations exploiting the code tridimensional feature provide an idea of the behavior of induced signal amplitude when only the primary coil (**S**_**C1**_) is used in the measurement. This decay is denoted by the function f_m_(**d**), which can be fitted exponentially. The amplitude of the induced voltage V_1_ in the **S**_**C1**_ coil is thus given by *V* ^0^_1_ *f*_*m*_(*d*), where in *V* ^0^_1_ is V_1_ at d = 0 mm. In other words, f_m_(**d**) directly shows the decay of the signal amplitude of **S**_**C1**_ coil in a circuit without the secondary coupling coil (**S**_**C2**_).

Coil coupling was also simulated using FEMM 4.2 software. In this situation, one coil was fed with a current of 1 Amp and the normal magnetic flux was assessed in the second one as a function of distance ***d***. Figure 2 presents the ratio (Φ_12_/Φ_11_) between the flux Φ_12_ created by coil **S**_**C1**_ in the coil **S**_**C2**_ as well as the flux Φ_11_, generated by **S**_**C1**_ itself. The flux Φ_12_ is related to mutual inductance M_12_ and the ratio Φ_12_/Φ_11_ is the coupling factor “k”. The flux Φ_11_ corresponds to self-inductance L_1_ or, for Φ_22_, L_2_. As L_1_ = L_2_ = L in this study, M_12_ = k (L_1_.L_2_)^1/2^ = k.L. Because of the magnetic field linearity generated by these coils, the self-inductance is directly obtained when the current is set to 1 Amp.

Figure 2 also shows that the fluxes of magnetized disks decay like the flux created by one coil in the other one. This happens because the homogeneously magnetized disk can be replaced by the magnetization current density, what makes the magnetic field of the disk equals to that of a coil with one turn.

**Figure 3:**
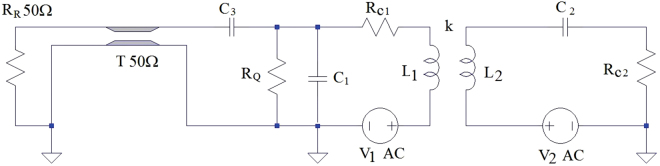
Electronic schematics applied for inductive coil coupling.

Circuitries for excitation/detection of coupling coil regarding both high and 50 Ω impedances may be found in the literature^15–18^. The circuits chosen in the experiment reported here are illustrated in Fig. 3. If one considers only the physically connected left side, it would be a common circuit typically used in NMR devices, applying tuning and matching capacitors (C_2_ and C_3_) as well as R_Q_ resistor for circuit quality factor (Q) adjustment. T means the coaxial cable (50 Ω) and the output voltage is on R_R_. Rc indicates the resistances for both coils. The entire circuit was then used for inductive coupling. It also consisted of capacitors for impedance matching and resonance frequency tuning.

Since the voltage induced in the coil **S**_**C1**_ is not negligible, the voltage source V_1_ was included in the circuit topology in addition to V_2_, which is induced in the coil **S**_**C2**_. These circuits were analyzed through *LTspice* code^19^. Although there was not a rigorous modeling taking into account the exact coil geometries, copper shielding, the ferromagnetic influence of the carbon steel base magnet, and the dynamic analyses, the gains associated to coupled coil signal and its quasi-constant behavior could be seen.

If the sub-circuit that contains the **S**_**C1**_ coil has a high impedance, as the **S**_**C1**_ coil terminals are open, the tuning is obtained by **C**_**2**_ capacitor and the mutual inductance does not influence the current in the coupled circuit. The maximum current I_2_ is given by V_2_/R_2_ at the resonance frequency (w_r_). In this situation, the voltage between the **S**_**C1**_ coil terminals (V_0c_) is then the voltage of the coupled coils subtracting the voltage directly induced by the nuclear spins in **S**_**C1**_, since they produce opposite magnetic fluxes. Thus, V_0c_ can be expressed by Eq. 1.

1 $${V}_{0c}=M{w}_{r}\frac{{V}_{2}}{{R}_{2}}-{f}_{m}(d){V}_{2}=[\frac{Lk{w}_{r}}{{R}_{2}}-{f}_{m}(d)]{V}_{2}$$

On the other hand, the voltage V_0_ of the uncoupled circuit is obtained by Eq. 2 and the gain is the ratio V_0c_/V_0_.

2 $${V}_{0}=-{f}_{m}(d){V}_{2}$$

### Intensity of the NMR signal versus sample distance (d) from the sensor

**Figure 4:**
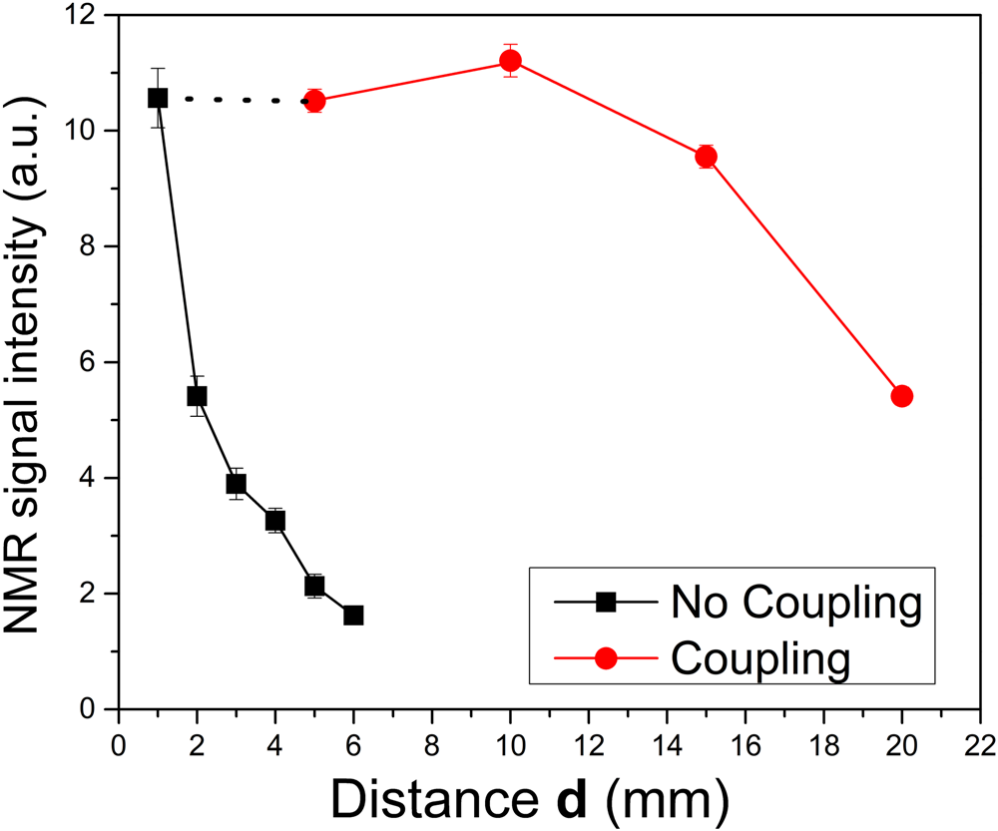
Maximum spin-echoes intensities at different distances **d** between the oil sample and the **S**_**C1**_ coil using the inductive coupled **S**_**C2**_ coil (red circles) and only **S**_**C1**_ coil (black squares).

To evaluate the improvement of signal intensity due to the implementation of a secondary inductive coil coupled to the UNMR sensor, NMR spin echoes of a thin automotive lubricant oil layer were recorded at different distances **d** from the sensor surface using surface coil and the wireless inductive coupled coil. **d** was varied through the sequential addition of microscopy glass sliders (1 mm in thickness) between the sensor and the sample. It is worth mentioning that, for the inductive coupled experiments, **d** reflects the distance between **Sc**_**1**_ and **Sc**_**2**_ coils, being the sample positioned right above the **Sc**_**2**_ coil. Figure 4 shows the maximum intensities of the NMR echoes recorded at different distances **d**.

As evidenced in Fig. 4, the intensities of the NMR signals using the surface coil **Sc**_**1**_ (squares) and the remote coil **Sc**_**2**_ (circles) are remarkably different at distances from the sensor higher than 2 mm. At approximately 6 mm, the signal resulting from **Sc**_**1**_ was within the detection limit and presented a SNR equal to 3, whereas the intensity measured at the same distance with **Sc**_**2**_ was identical do the **Sc**_**1**_ on the surface. The intensity of the signal at 20 mm using the wireless remote coil was equal to that at 2 mm using the surface coil. This is equivalent to a one order of magnitude enhancement in signal intensity, indicating the greater advantage of this coupling. Although this advantage is not universal, we can envisage numerous applications for the wireless remote inductive coupled coil, which can boost the use of remote NMR.

### Monitoring plaster curing

**Figure 5:**
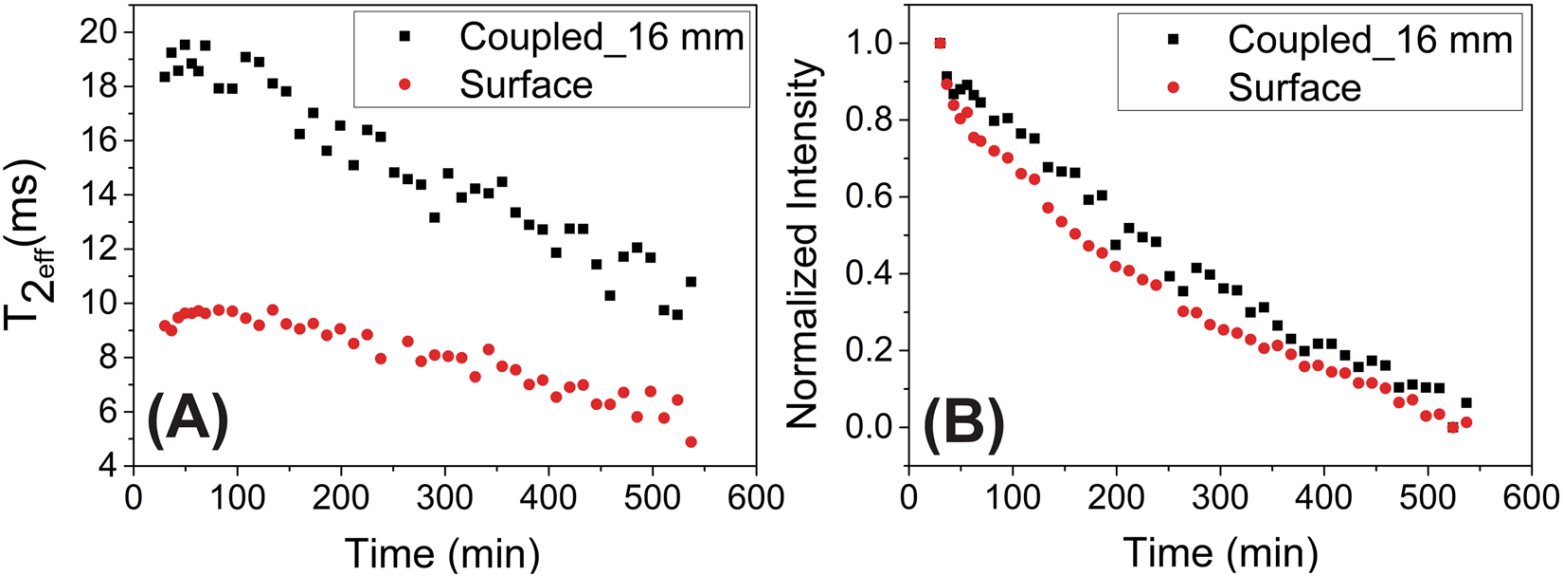
Evolution of the NMR signal acquired by CPMG pulse sequence during plaster curing. (**A**) is the determined T_2eff_ and (**B**) is the normalized maximum intensity of the first CPMG echo. Both data are plotted over curing time. The squares are data from the inductive coupling of **S**_**c2**_ (embedded in the plaster cube and located 16 mm from the NMR sensor surface), while the circles are data from the common set-up (sample located close to the sensor surface, with **d** = 2 mm).

The secondary coil was also used to monitor the curing process of plaster. Figure 5a shows the evolution of the maximum signal intensity of the first echo, while Fig. 5b presents the behavior of effective transverse relaxation time (**T**_**2eff**_) recorded by the CPMG pulse sequence of a plaster block (40 × 40 × 35 mm^3^) as a function of curing time. The circles denote the measurements made 2 mm away from the sensor surface (**d** = 2 mm) using only the primary coil. The squares are measurements made using the secondary coil embedded in the plaster cube as well as inductive coupled to the primary coil. It was located 16 mm from the magnet surface.

Even though discussing mass transport and water mobility (curing process) on a time course in not the goal of this work, one may observe in Fig. 5b that the signal intensity related to water content decays faster on the surface than in the center of the plaster block. The difference between such positions was not larger because the glass sliders used to protect the **S**_**c1**_ coil limited water evaporation. Water loss during the experiments was approximately 25%.

Larger differences between **T**_**2eff**_ were observed in the two positions (Fig. 5b). The lower **T**_**2eff**_ values on the surface can be explained by fast decay of CPMG signal caused by water diffusion in the presence of larger magnetic field inhomogeneity at the magnet surface^4^. At 16 mm, the magnetic field inhomogeneity is smaller than on the magnet surface and the effect of water diffusion is less pronounced. The smaller decay of **T**_**2eff**_ values on the surface than at 16 mm can be explained by the reduced water diffusion due to hydration process that takes place concomitantly to water evaporation^20^.

## Discussion

In summary, a simple, efficient means of enhancing the detection distance of small UNMR sensors based on wireless inductive coupling sensor, involving neither coils featuring sophisticated designs nor larger magnets and coils, was demonstrated. The proposed configurations increased the detection distance by more than one order of magnitude, opening new opportunities for field applications of UNMR sensor. Although this advantage is not universal, several applications of the wireless remote inductive coupled coil may be envisaged, boosting the use of remote NMR. Some potential applications are listed below:

- Monitoring chemical reactions that need to be isolated from the NMR sensor. The remote wireless sensor can also be applied to reduce the effect of magnetic field on electrochemical reactions^21^, as the electrochemical cell can be positioned in an area of low magnetic field.
- Biomedical applications, in which the **Sc**_**2**_ coils could be applied as bandages on specific regions of the body to evaluate the evolution of bone or soft tissue lesions caused by traumatic and infectious processes or even tumoral lesions.
- Materials Engineering, to monitor polymerization reactions either in laboratory or pilot scales as well as in manufactured products. Civil Engineering, to monitor curing and degradation processes of cement. In the latter, embedding **Sc**_**2**_ coils into a concrete structure could provide valuable internal chemical and physical information concerning the sample.

Moreover, the simple **S**_**C2**_ design, built on the thin-coppered print circuit board, keeps the instrumentation costs low, which creates the possibility to make disposable coils depending upon the application.

## Methods

### Home-built UNMR sensor

A more detailed description of the UNMR sensor construction can be found elsewhere^22^. Basically, it was built using four axially magnetized NdFeB alloy blocks (2.54 × 2.54 × 1.27 cm^3^) mounted on a carbon steel yoke base. Each pole was constructed with two blocks with the same orientation and separated by a 2-mm gap. The separation between the two poles was 14 mm^6,22,23^. The coil probe (**Sc**_**1**_) was built in 20 awg copper wire with 9 turns and having an external diameter equal to 20 mm. Matching and tuning variable capacitors (**Cs**) ranging from 5 to 25 pf and from 5 to 50 pf, respectively, were used to adjust the probe between 25.550 MHz (0.6 T on sensor surface) and 10.650 MHz (0.25 T, 20 mm above sensor surface), where the system showed Q values around 50. For frequencies lower than 24.000 MHz, chip ceramic capacitors up to 300 pf were added in parallel to tune the circuit at the desired frequency. The UNMR sensor was connected to a NMR console CAT-100 (*Tecmag*, Houston, USA), a 3205 AMT power amplifier, and an AU 1114 preamplifier.

### Secondary coil fabrication

**Figure 6:**
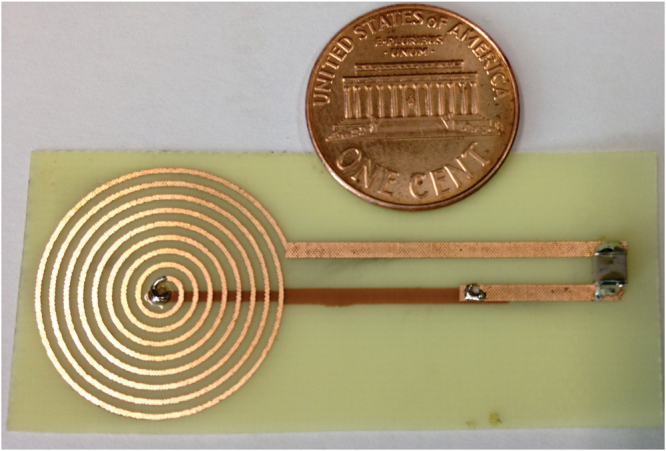
Secondary coil built on a double-sided coppered printed circuit board. A penny is shown for size comparison. The chip ceramic capacitor soldered to the coil can be seen on the right-end edge.

The wireless remote coil **Sc**_**2**_ was etched in a 0.2-mm thick, double-sided, coppered printed circuit board (55 × 25 mm^2^). The resonance circuit was tuned to each frequency by a specific chip ceramic capacitor (**C**_**s2**_). The **S**_**C2**_ coil had 9 turns with an external diameter of 20 mm. As previously stated, the chip capacitor soldered to it was intended to keep the coupled system working on the over coupling regime, *e.g*. for 11.010 MHz at 20 mm, whereas the primary coil was set at 10.912 MHz, the secondary coil was set at 11.287 MHz. Figure 6 shows a picture of the secondary coil.

### NMR experiments

The intensities of the NMR echo signals were acquired using a spin echo sequence with 2-μs-long RF pulses separated by a time τ of 512 μs and averaged with 200 scans. The intensities of the echo signals were measured with an automotive lubricant oil sample (0.5-mm-thick layer). For the analyses without the remote coil, the sample distance to the sensor varied up to 6 mm, while for the remote coil it ranged up to 20 mm. CPMG pulse sequence was used to monitor the plaster curing process using 2-μs-long RF pulses separated by a time τ of 500 μs, 1200 refocusing pulses, and an average of 300 scans.

### Plaster curing experiment

The plaster was bought in a neighborhood market as plaster of Paris powder. It was previously prepared in a beaker by mixing powder and tap water at a 1:0.8 weight ratio. Then, it was transferred to an acrylic box of 40 × 40 × 35 mm^3^ (l x w x h) with a 1-mm glass slider at the bottle, for molding and setting on the UNMR sensor. To monitor the curing process on the surface, a sample of freshly prepared plaster was placed on the surface of the **Sc**_**1**_ coil. For remote analyses, the **Sc**_**2**_ coil was embedded in the center of the plaster. Subsequently, 43 CPMG experiments were performed during 9 hours. To accelerate the curing process, a fan was placed 50 cm away from the experimental set-up and the room temperature was maintained at 25 ± 1 °C.
